# Case Report of Cerebral Sinus Thrombosis Related to Immune Thrombotic Thrombocytopenia Following Administration of ChAdOx1 nCoV-19 for Vaccination against COVID-19

**DOI:** 10.3390/life12020168

**Published:** 2022-01-24

**Authors:** Wojciech Szypowski, Aleksander Dębiec, Jarosław Świstak, Maciej Nowocień, Piotr Rzepecki, Marcin Możański, Jacek Staszewski, Adam Stępień

**Affiliations:** 1 Clinic of Neurology, Military Institute of Medicine, 04-141 Warsaw, Poland; wszypowski@wim.mil.pl (W.S.); jswistak@wim.mil.pl (J.Ś.); jstaszewski@wim.mil.pl (J.S.); astepien@wim.mil.pl (A.S.); 2 Medical Diagnostic Imaging, Military Institute of Medicine, 04-141 Warsaw, Poland; mnowocien@wim.mil.pl; 3 Clinic of Haematology, Military Institute of Medicine, 04-141 Warsaw, Poland; przepecki@wim.mil.pl; 4 Clinic of Anaesthesiology and Intensive Care, Military Institute of Medicine, 04-141 Warsaw, Poland; mmozanski@wim.mil.pl

**Keywords:** COVID-19, COVID-19 vaccine, CVST, VITT, thrombocytopenia

## Abstract

Vaccine-induced immune thrombotic thrombocytopenia (VITT) with cerebral venous sinus thrombosis (CVST) has been recently reported after vaccination against severe acute respiratory syndrome coronavirus 2 (SARS-CoV-2). We present a case of a patient with fulminant CVST and thrombocytopenia after receiving the ChAdOx1 nCoV-19 vaccine. Although the patient received immediate anticoagulant and intravenous immune globulin treatment, he died within 24 h after hospital admission. VITT and CVST are rare conditions; however, the course may be fatal. Therefore, clinicians should be familiarized with the clinical and laboratory features of VITT.

## 1. Introduction

COVID-19 vaccines play a significant, important and protective role in the management of the global pandemic of coronavirus disease. COVID-19 vaccines were approved for use after evaluation in clinical trials, and different national surveillance programs supporting vaccinations are ongoing. However, extremely rare cases of cerebral venous sinus thrombosis (CVST) and thrombocytopenia following COVID-19 vaccines have been observed since the beginning of 2021 in a small number of individuals. A possible immune response mechanism for the development of thrombocytopenia and thrombosis after receiving ChADOx1 nCOV-19 has been developed, and researchers categorize that syndrome as vaccine-induced immune thrombotic thrombocytopenia (VITT) [1].

The exact incidence of VITT is unknown, but the condition is probably very rare. Despite its low incidence, mass vaccination has resulted in an increased risk of developing this condition; therefore, clinicians need to be aware of its presenting features and appropriate evaluation and management. Here, we describe a patient with CVST and thrombocytopenia with a fulminant course admitted to a public community hospital.

## 2. The Case

**Figure 1:**
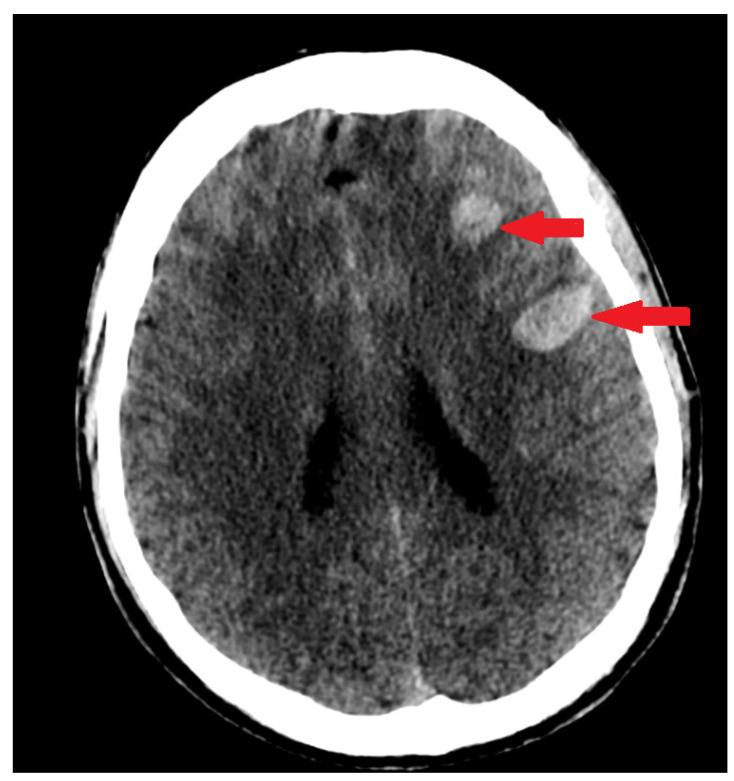
Axial CT scan without contrast enhancement. Red arrows show two spots of unilateral typical deep cortical venous hemorrhages with minor surrounding oedema in the left frontal lobe of the brain. The frontal hemorrhage spot crosses the borders of arterial brain vascular territories.

**Figure 2:**
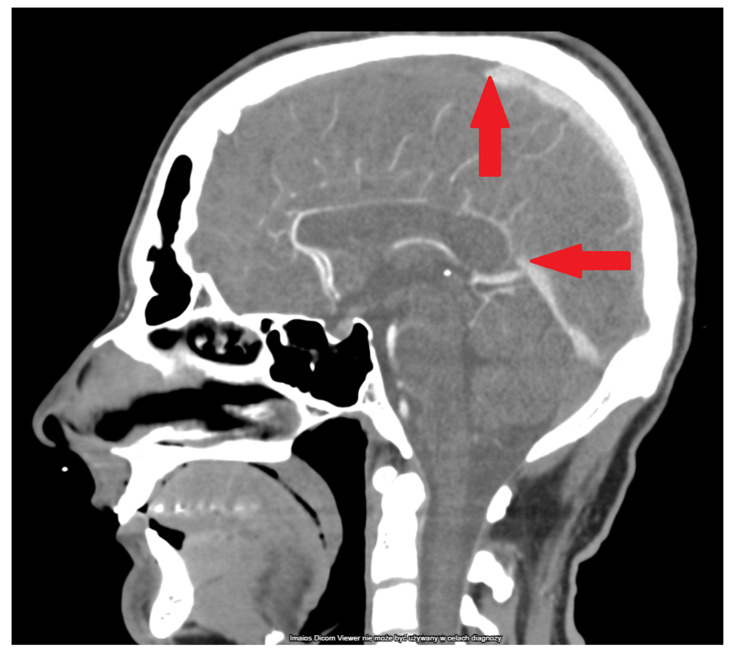
Sagittal CT scan with contrast enhancement. Red arrows point to the sharply demarcated end of venous thrombus in the superior sagittal sinus and in the great cerebral vein (of Galen). Additionally, the inferior sagittal sinus is not filled with contrast, indicating thrombosis.

A 28-year-old previously healthy man was admitted to the Emergency Department of the Military Institute of Medicine in Warsaw with prolonged impaired consciousness following a first in-life episode of generalized seizure. He developed flu-like symptoms a few hours after the first dose of the COVID-19 vaccine (AstraZeneca, University of Oxford, and Serum Institute of India; ChAdOx1 nCoV-19). Following four days of health improvement, he again developed severe persistent headache and fever at Day 5 post vaccination. On admission to the hospital (7 days after vaccination), the patient was somnolent and had severe aphasia and left side paresis. The other findings upon physical examination were normal. Computed tomography imaging (CT) revealed acute cortical hemorrhage in the left frontal lobe and subarachnoid hemorrhage over the right frontal lobe and along the falx cerebri (Figure 1). The CT venogram showed extensive thrombosis of the superior sagittal sinus along the entire length of the frontal lobes and the non-contrast inferior sagittal sinus of the brain together with clotted right and left Trolard veins (Figure 2). Additional examinations revealed thrombocytopenia (39,000/µL) and elevated levels of D-dimer (125,780 µgFEU/L) and CRP (2.3 mg/dL) (Table 1). The patient had negative PCR COVID test results. Routine chest X-ray was normal. His past medical history and family history were unremarkable, and there was no personal or family history of thromboembolic events. No allergic reactions were reported in previous vaccinations. No known clinical risk factors for thrombosis or cerebral venous sinus thrombosis (CVST) could be identified. Following consultation by a neurosurgeon who qualified the patient for conservative treatment, the patient was admitted to the neuro-intensive care unit. Due to brain hemorrhage and thrombocytopenia, the patient received an intermediate dose of low-molecular-weight heparin (LMWH; 1 mg/kg s.c.). The patient deteriorated 6 h following admission and developed coma (Glasgow Coma Scale (GCS) 3) and acute respiratory failure. He was intubated, and mechanical ventilation was introduced. Following hematological consultation, the patient was suspected to have vaccine-induced immune thrombotic thrombocytopenia, and he was urgently administered fondaparinux (7.5 mg s.c. once daily) and intravenous immune globulin (1 g/kg/day). Anti-PF4 antibodies could not be assessed due to a technical inability to perform that laboratory test in the hospital.

**Figure 3:**
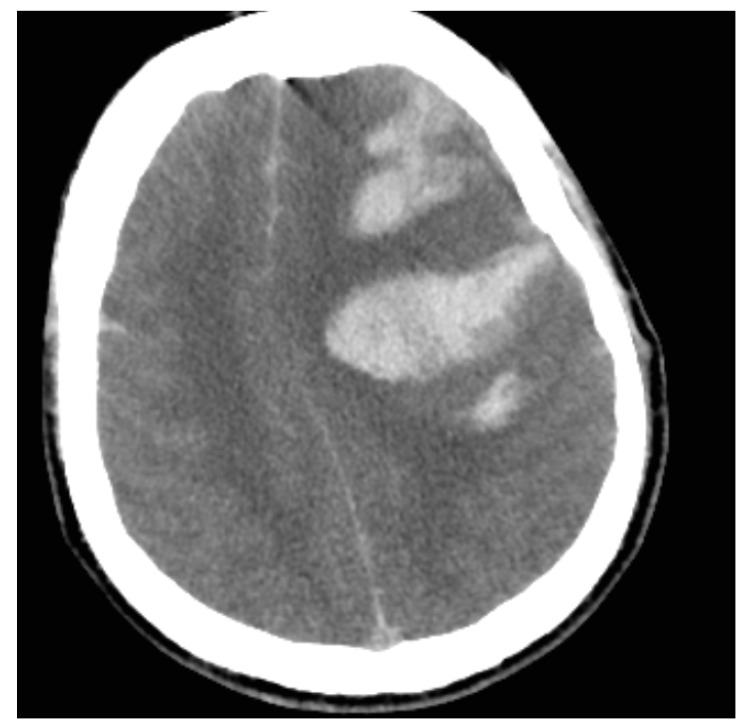
Axial CT scan. Massive left frontal lobe hemorrhage with brain swelling. Note the fluid-fluid levels, the subarachnoid hemorrhage and the heterogeneous appearance of all hematomas.

The control CT scan showed a massive left intracerebral hematoma along with no contrast flow in distal branches of the brain arteries, and control D-dimer levels showed an increasing trend despite treatment (Figure 3, Table 1). The patient was again consulted by the neurosurgeon but was not qualified for neurosurgical intervention due to clinical and radiological signs of massive and irreversible brain damage and suspicion of brain death. He developed hemodynamic failure and required administration of catecholamines (noradrenaline) and stimulation with diuresis. Brain death was confirmed by subtraction angiography of the brain, which was performed after 12 h of observation and revealed the absence of intracranial blood flow. An autopsy was not performed. The probable postvaccination reaction has been reported to the local County Sanitary and Epidemiological Station.

CT of the head on admission (Figure 1): An acute parenchymal hemorrhage in the left frontal lobe (26 × 14 × 40 mm). SAH over the right frontal lobe and cerebral falx.

CT venography on admission (Figure 2): Thrombosis of the superior and inferior sagittal sinuses. Bilateral thrombus in the superior anastomotic (Trolard) vein. Homogeneous intraluminal signal of cerebral arteries.

Thoracic X-ray on admission: Lungs appear normal with no focal densities and pulmonary congestion.

CT of the head after 6 h (Figure 3): An acute parenchymal hemorrhage in the left frontal lobe (58 × 47 × 52 mm). Progression of SAH over the right frontal lobe and cerebral falx. Intraventricular hemorrhage (the left lateral ventricle). Cerebral oedema appears bilaterally with compression of basal cisterns and a 15-mm midline shift to the right. Uncal herniation is noted.

DSA after 24 h (not shown): No cerebral circulation and lack of venous return.

ACA-anterior cerebral artery, ALT-alanine aminotransferase, APTT-activated partial thromboplastin time, AST-aspartate aminotransferase, CRP-C-reactive protein, CT-computed tomography, DSA-digital subtraction angiography, eGFR-estimated glomerular filtration rate, ICA-internal carotid artery, INR-international normalized ratio, SAH-subarachnoid hemorrhage, MCA-middle cerebral artery, PT-prothrombin time, WBC-white blood cells

## 3. Discussion

We describe a patient presenting with sudden progressive neurological symptoms due to CVST with massive intracerebral hemorrhage and concomitant thrombocytopenia 7 days after vaccination with ChAdOx1 nCoV-19. Although the patient received urgent anticoagulation followed by IVIg, he unfortunately developed massive and fatal intracranial bleeding related to progressive CVST. Some similar cases have been published, but the awareness of physicians regarding the clinical picture of the disease, its diagnosis and treatment taking into account its possible fulminant course (resulting in death within hours after the onset of acute syndromes) should be increased. To the best of our knowledge, our case report is the first case reported in Poland.

This characteristic pattern of CVST in combination with thrombocytopenia is the most prominent presentation of VITT, a recently described possible complication after vaccination with adenoviral vector-based vaccines [2]. This condition has been observed since the beginning of 2021 in a very small number of individuals who received ChAdOx1 nCoV-19 or Ad26.COV2.S vaccine (Janssen Pharmaceuticals, Beerse, Belgium; Johnson & Johnson, New Brunswick, NJ, USA); however, no direct link between vaccination and thrombocytopenia has been proven. Only a hypothesis regarding a condition similar to heparin-induced thrombocytopenia (HIT) has been proposed.

### 3.1. Pathophysiology

VITT is caused by antibodies against platelet factor 4 (PF4) that activate platelets via low affinity platelet FcγIIa receptors, resulting in marked stimulation of the coagulation system and clinically significant thromboembolic complications. The mechanism responsible for the development of new antibodies or immune stimulation of preexisting antibodies has not been clarified to date. Autopsy studies have demonstrated catastrophic venous thrombosis involving multiple large and small vessels resulting in pulmonary embolism, deep vein thrombosis and other unusual sites, including the splanchnic veins, adrenal veins and the ophthalmic and cerebral veins [3].

### 3.2. Prevalence and Mortality

The worldwide incidence of VITT is unknown. The highest incidence was noted in Norway, in which five cases were reported from among approximately 130,000 individuals vaccinated with ChAdOx1 nCoV-19, suggesting an incidence of 1 in 26,000 [1]. However, despite over 30 million fully vaccinated people worldwide, to the best of our knowledge, less than 50 cases of CVST with thrombocytopenia after COVID-19 vaccination have been published, and 187 cases were reported in the European Union according to EudraVigilance giving six cases of CVST per 1 million vaccinated people [4,5].

According to Global Advisory Committee on Vaccine Safety (GACVS) data, VITT symptoms occur in approximately 4 to 6 people out of every million vaccinated with the AstraZeneca vaccine (as of 15 July 2021), and the frequency varies based on age, sex and geographical location [6]. Regarding the Janssen vaccine (as of 7 May 2021), the US Food and Drug Administration and the Centers for Disease Control and Prevention reviewed 28 reports of VITT of more than 8 million vaccinated individuals [7]. The most recent information from the UK suggests an incidence of 20.3 per million doses in people between 18 and 49 years for the AstraZeneca vaccine compared to 10.9 per million doses in those aged ≥50 years [8]. The European Medicines Agency (EMA) stated that the benefits of vaccination outweigh any risks of side effects in relation to the vaccine after reporting 62 cases of CVST and 24 cases of splanchnic vein thrombosis, 18 of which were fatal [9]. According to the WHO, although it is possible that a causal link exists between the vaccine and VITT, more data are needed [10].

The incidence is far lower than that of CVST following COVID-19, which has an approximately seven-fold greater incidence (39 per million people) [11]. Despite the rare frequency, CVST after COVID-19 vaccination is believed to be a matter of great importance due to a high complication rate and mortality compared to idiopathic CVST, which exceeds 50% [12,13,14].

### 3.3. Risk Factors

Our case is interesting because the majority (75%) of CVSTs after vaccination have been reported thus far in women, and the course was fatal within hours after acute symptoms [15]. Both the EuduraViligance report and published data from other countries indicate an increased risk, especially among females older than 45 years. On the other hand, this gender bias is also noted in cases with idiopathic CVST. Analysis of the risk factor profile demonstrated that only 11% of CVST patients have typical risk factors for oral contraception, hereditary thrombophilia, puerperium or tumors [8]. Initial reports suggest that individuals with VITT are younger than those with idiopathic CVST (younger than 55 years).

### 3.4. Symptomatology

High variance in CVST symptoms leads to difficulties in the diagnostic process. In patients with CVST following COVID-19 vaccination, most of the symptoms are reported early after the first dose of ChAdOx1 nCov-1, usually within the first 7–9 days. Almost all published cases described headaches among the first symptoms. Sinus thrombosis leads to sinus occlusion and backflow of blood into venules and capillaries, resulting in increased local pressure and eventually resulting in massive cerebral oedema [16]. Seizures are common in the acute phase of CVST and are typically related to superior sagittal sinus thrombosis or hemorrhagic supratentorial lesions [17,18]. Both venous and arterial thromboses have been described, and CVST appears to be the most common site of thrombosis in some series [19]. This condition may lead to venous congestion, cerebral oedema, and ischemic and/or hemorrhagic stroke. Intracerebral hemorrhage or subarachnoid hemorrhage (SAH) due to CVST was reported in approximately 50% of patients [20].

### 3.5. Imaging Studies

Urgent neuroimaging with cranial CT and CT or MRI venography is recommended for the diagnosis of CVST [21]. All brain dural sinuses and veins can be affected, but the most common CVSTs are the superior sagittal sinus, straight sinus, transverse sinus, sigmoid sinus, and cavernous sinus. Most commonly, CT without contrast enhancement is the first step of diagnostic imaging, and CVST without brain hemorrhage or oedema can be a subtle finding on CT images. It is worth emphasizing that some case reports described a lack of typical imaging changes in the cerebral sinus despite clinical symptoms [22]. Potential findings in unenhanced CT include hyperdense or occasionally thickened sinuses (especially Trolard or Labbé veins) and typical images of CVST hemorrhage: round or oval, deep cortical hemorrhage with partial sparing of near cortical tissue and subarachnoid hemorrhage. CVST hemorrhage or infarct can cross the borders of arterial brain vascular territories [23]. Enhanced CT or MR venography shows a sinus filling defect with an empty delta sign, gyral enhancement, and a prominent intramedullary vein. Therefore, sinus thrombosis should not be excluded exclusively on the basis of the results of imaging studies [24].

### 3.6. Laboratory Tests

Cerebral venous sinus thrombosis can be associated with VITT, the course of which is similar to autoimmune heparin-induced thrombocytopenia (HIT). Although an association with the ChAdOx1 nCoV-19 vaccine seems obvious, the exact cause of this immune reaction is unclear.

Testing for PF-4 antibodies might be useful as a confirmatory test, but a positive anti-PF4 test without other abnormalities is insufficient to make the diagnosis [25]. The initial and subsequent laboratory testing recommendations differ between guidelines. Standard tests that should be performed include platelet count, prothrombin time, activated partial thromboplastin time, fibrinogen level measured by the Clauss method, and D-dimer level. Clinicians should become familiarized with their laboratory methodology, reporting units and cut-offs because some treatment decisions are based on these factors. In addition, direct extrapolation may not be straightforward. D-dimer levels should be reported in FEU (fibrinogen equivalent unit); the normal level is less than 500 FEU (equivalent to <250 ng per milliliter). Anti-PF4 antibodies should be detected using the HIT ELISA method, as chemiluminescence assays with high sensitivity for HIT were found to be unreliable in detecting anti-PF4 antibodies in patients with VITT [19].

Case definition criteria for vaccine-induced immune thrombocytopenia and thrombosis, according to an Expert Hematology Panel, are presented in Table 2 [19].

It should be stressed that to ascertain the possibility that the vaccine could be considered a trigger for adverse events following immunization (AEFI), each hypothesis regarding other causes should be excluded [26]. Although it is difficult to confirm the causality between the ChAdOx1 nCoV-19 vaccine and CVST, our case shows a temporal association between the two events (the third level of analysis). Similar cases with CVST following vaccination have also been described in the literature, and the existence of other causes of CVST, such as undiagnosed SARS-CoV-2 infection, chronic inflammatory diseases, and obesity, was excluded in the presented patient. We could not however exclude thrombo-filia or vaccine preparation mistakes and administration errors; however, the latter would be extremely unlikely. The presented patient fulfilled the criteria for probable VITT (highly elevated D-dimers, timing of the symptoms related to vaccination, CVST and thrombocytopenia, and no assessment of anti-PF4 antibodies). Based on the clinical and radiological picture, we recognized CVST, but we could not exclude thrombosis in other sites. Data from the literature suggest that diffuse and severe thrombosis in VITT can be common in unusual sites, including the lungs, liver, heart, aortic vasa vasorum, kidneys and choroid plexuses [27]. Although the clinical features of the subject resemble well-described heparin-induced thrombocytopenia (HIT), no high-level scientific evidence has been reported to confirm the causal association to date [28]. Despite an association between PF-4 antibodies and VITT, further studies are needed to assess whether these antibodies are induced by vaccines cross-reacting with PF4 and platelets or strong inflammatory stimuli of the vaccine itself.

### 3.7. Treatment

The cornerstone in CVST treatment in the acute phase is anticoagulant therapy despite cerebral hemorrhage [15]. VITT is a potentially life-threatening disorder. However, as VITT with or without CVST are newly described syndromes, all guidelines are based on recommendations for HIT treatment and include intravenous immunoglobulins (0.5–1 g/kg daily for two days), steroids (included intravenous methylprednisolone, oral or intravenous dexamethasone, and oral prednisolone, for example prednisone 1–2 mg/kg), and anticoagulation based on non-heparin agents (fondaparinux, argatroban, bivalirudin, or direct oral anticoagulant, e.g., apixaban, rivaroxaban). In our case, the patient was initially treated with LMWH before the final diagnosis was established. It is unknown whether heparin is effective or deleterious in individuals with VITT; however, preliminary in vitro studies suggest that heparin is not harmful. It may be therefore reasonable to avoid heparin in cases of diagnostic uncertainty in which delayed or spontaneous HIT remains possible. In a series of 220 individuals with definite or probably VITT, heparin did not appear to be harmful. However, this cohort was heavily biased towards presentations earlier in the pandemic when the syndrome was unrecognized, and outcomes may have been adversely affected by delays in diagnosis [19]. The mortality associated with VITT was highest among patients with thrombocytopenia and intracerebral hemorrhage.

Early plasma exchange can be considered in cases of low platelet count despite intravenous immunoglobulin and steroid therapy. Patients with a baseline platelet count of less than 30,000/mm³ may receive a platelet transfusion, typically in preparation for neurosurgery or for anticoagulation. On the other hand, there is some concern that platelet transfusions may cause worsening thrombosis, a theoretical risk based on extrapolation from other conditions.

The length of acute illness in VITT is not known, and thrombocytopenia can persist for days to weeks. Intracranial hemorrhage and SAH combined with CVST are associated with poorer prognosis, and there are currently no strong recommendations regarding thrombectomy use in these conditions [29]. However, despite this, patients who deteriorate rapidly and do not respond to standard therapy can benefit from interventional services, such as intra-sinus thrombolysis or mechanical thrombectomy. Previous studies have shown that mechanical thrombectomy alone and combined with intra-sinus thrombolisis should be considered in severe cases and is reasonably safe. However, further controlled studies are required to confirm the efficacy and validity of these methods [30,31].

In one published randomized controlled study (Thrombolysis or Anticoagulation for Cerebral venous Thrombosis) comparing endovascular thrombolysis with standard therapeutic anticoagulation, the trial was stopped because the proportion of patients with the primary outcome of a modified Rankin Scale of 0–1 was similar in both treatment groups [32].

### 3.8. Prognosis

Prognosis varies. Based on a series of patients with definite or probable VITT, the mortality rate was 22%, and CVST with or without severe hemostatic abnormalities was established to be a major risk factor for death. In patients with impending herniation, decompressive hemicraniectomy (DH) can be life-saving; however, the quality of the evidence on this intervention is low because no randomized controlled trials have been performed thus far [15]. Rapid assessment of DH, especially at admission, is crucial because the progression of CVST can be fulminant, as noted in the presented case.

## 4. Conclusions

COVID-19 vaccines provide strong protection against serious illness, hospitalization and death. Encouraging data regarding their efficacy and safety are available from both clinical trials and different country surveillance programs. More serious or long-lasting side effects of COVID-19 vaccines are very rare. CVST events have been reported after vaccination with adenoviral COVID-19 vector vaccines. Although they are extremely rare, the course, as described in the current report, may be fulminant; therefore, clinicians should be familiarized regarding the clinical and laboratory features of VITT as well as recommended methods of treatment [15].

## Figures and Tables

**Table 1:** Blood and diagnostic imaging results.

Lab Finding	Admission	After 6 h	After 12 h
Platelet count (×10^9^/L)	39	92	65
Hemoglobin (g/dL)	14.7	15.5	16.8
WBC (×10^9^/L)	8.18	13.44	23.13
APTT (s)	42.1	61.6	51.2
PT (s)	12.9	12.9	13.6
INR	1.14	1.14	1.21
D-Dimer (µgFEU/L)	125,780	153,660	157,660
Fibrinogen (mg/dL)	-	183	314
AST	-	38	99
ALT	-	34	71
Creatinine (mg/dL)	1.0	1.3	2.0
eGFR (ml/min/1.73 m^2^)	>90	>90	42
Urea (mg/dL)	33	32	54
CRP (mg/dL)	2.3	5.2	-

**Table 2:** Case definition criteria for Vaccine-induced Immune Thrombocytopenia and Thrombosis (VITT) according to an Expert Hematology Panel [19].

Type of VITT	Description
Definite VITT	All five of the following criteria: onset of symptoms 5–30 days after vaccination against SARS-CoV-2 (or ≤42 days in patients with isolated deep-vein thrombosis or pulmonary embolism) presence of thrombosis thrombocytopenia (platelet count <150,000 per cubic millimetre) D-dimer level >4000 FEU positive anti-PF4 antibodies on ELISA
Probable VITT	D-dimer level >4000 FEU but one criterion not met (timing, thrombosis, thrombocytopenia, or anti-PF4 antibodies) or D-dimer level unknown or 2000–4000 FEU and all other criteria met
Possible VITT	D-dimer level unknown or 2000–4000 FEU with one other criterion not met, or two other criteria not met (timing, thrombosis, thrombocytopenia, or anti-PF4 antibodies)
Unlikely VITT	platelet count <150,000 per cubic millimetre without thrombosis with D-dimer level <2000 FEU, or thrombosis with platelet count >150,000 per cubic millimetre and D-dimer level <2000 FEU regardless of anti-PF4 antibody result and alternative diagnosis more likely

ELISA denotes enzyme-linked immunosorbent assay; FEU, fibrinogen-equivalent unit; PF4, platelet factor 4; SARS-CoV-2, severe acute respiratory syndrome coronavirus 2.

## Data Availability

The data presented in this study are available on request from the corresponding author. The data are not publicly available due to an ongoing analysis.
